# Cerebral Venous Air Embolism: A Rare Clinical Challenge and Management Insights

**DOI:** 10.7759/cureus.73621

**Published:** 2024-11-13

**Authors:** Basheer Ahmed, Ahmed Musa, Arumugam Ravindrane, Muhammad M Meer

**Affiliations:** 1 Psychiatry, Palmer Community Hospital, Cumbria, Northumberland, Tyne and Wear NHS Foundation Trust, South Tyneside, GBR; 2 Radiology, Newcastle upon Tyne Hospitals NHS Foundation Trust, Newcastle, GBR; 3 Geriatric Medicine, Gateshead Health NHS Foundation Trust, Gateshead, GBR; 4 Acute Medicine, Northampton General Hospital, Northampton, GBR

**Keywords:** carotid artery imaging, cerebral venous embolism, chronic obstructive pulmonary disease, imaging diagnostic radiology in cerebral air embolism, ischemic lesion, neurovascular radiology

## Abstract

Cerebral air embolism (CAE) is a rare but life-threatening condition often associated with trauma, such as chest and skull injuries, which allow air to enter the venous system, as well as medical procedures and surgical interventions. It can occur during the insertion of peripheral cannulas or central midline catheters, following lung biopsy procedures, or during vascular surgeries, particularly those involving the head and neck region. CAE can also develop during the removal of central venous cannulas, as air may enter the bloodstream in the process. When air enters the bloodstream, it can travel to the cerebral blood vessels, where it may be trapped, forming bubbles that obstruct the blood flow. This blockage reduces oxygen supply to brain tissue, which can quickly lead to cell damage or ischemia if not resolved.

We present the case of a 62-year-old male with an infective exacerbation of chronic obstructive pulmonary disease who developed acute unilateral sensorimotor weakness several days following midline catheter insertion for a prolonged course of antibiotic administration. Prompt detection and intervention are essential in managing CAE to minimize risks and prevent permanent damage. The role of diagnostic radiology is essential in the rapid diagnosis and management of CAE. Imaging techniques such as computed tomography (CT) scans, carotid and cerebral angiograms, and magnetic resonance imaging (MRI) of the head are invaluable for assessing cerebral arteries and determining the extent of ischemic damage over time. They can also show signs of air trapped either in the venous or arterial system as the complexity of CAE is heightened by air emboli affecting various vascular regions, including the cerebral venous sinuses, requiring comprehensive imaging for accurate diagnosis and management. While CT of the brain is essential for immediate diagnosis, follow-up MRI scans provide detailed insights into the progression of ischemic changes that may result from CAE.

## Introduction

Cerebral air embolism (CAE) is a rare diagnostic finding, presenting as an adverse effect of different medical conditions and surgical interventions. CAE is reported with sudden-onset focal neurological symptoms, coma, epileptic seizures, and encephalopathy [1]. Fastidious treatment with hyperbaric oxygen has shown better neurological outcomes in this condition. CAE, while rare, is associated with an increased risk of mortality, with around a fifth of those afflicted succumbing to the disease [2].

When air enters the bloodstream, it can move freely into different routes such as pulmonary circulation, retrograde ascending into the cerebral venous system, or through a right-to-left shunt as in patent foramen ovale or septal defects. In retrograde ascending, it is usually noticed in a semi-upright or sitting position as air will enter backflow into the cerebral veins [3].

The venous vascular system can be compromised when air enters the circulation, which is a leading cause of venous air embolism. This typically occurs when a change in the pressure gradient promotes air entry into the circulation, with positive outside pressure and negative venous pressure permitting air entrance. In a systematic review, it was noted that most venous air embolism cases are iatrogenic, with approximately 50% resulting from surgical interventions and around 45% related to the insertion or removal of central venous catheters or peripheral intravenous (IV) lines [4]. Here, we report the case of a 62-year-old male with CAE following midline catheter insertion and provide a review of similar cases in the literature.

## Case presentation

A 62-year-old male with severe chronic obstructive pulmonary disease (COPD) requiring long-term oxygen therapy in the community was admitted to the respiratory department for exacerbation of COPD secondary to ongoing breathlessness requiring further oxygen administration and monitoring. After sputum samples showed *Pseudomonas* infection, he received ceftazidime (2 g three times a day). As the course of antibiotics was determined for two weeks, a midline catheter was inserted on the fourth day of admission.

**Figure 1:**
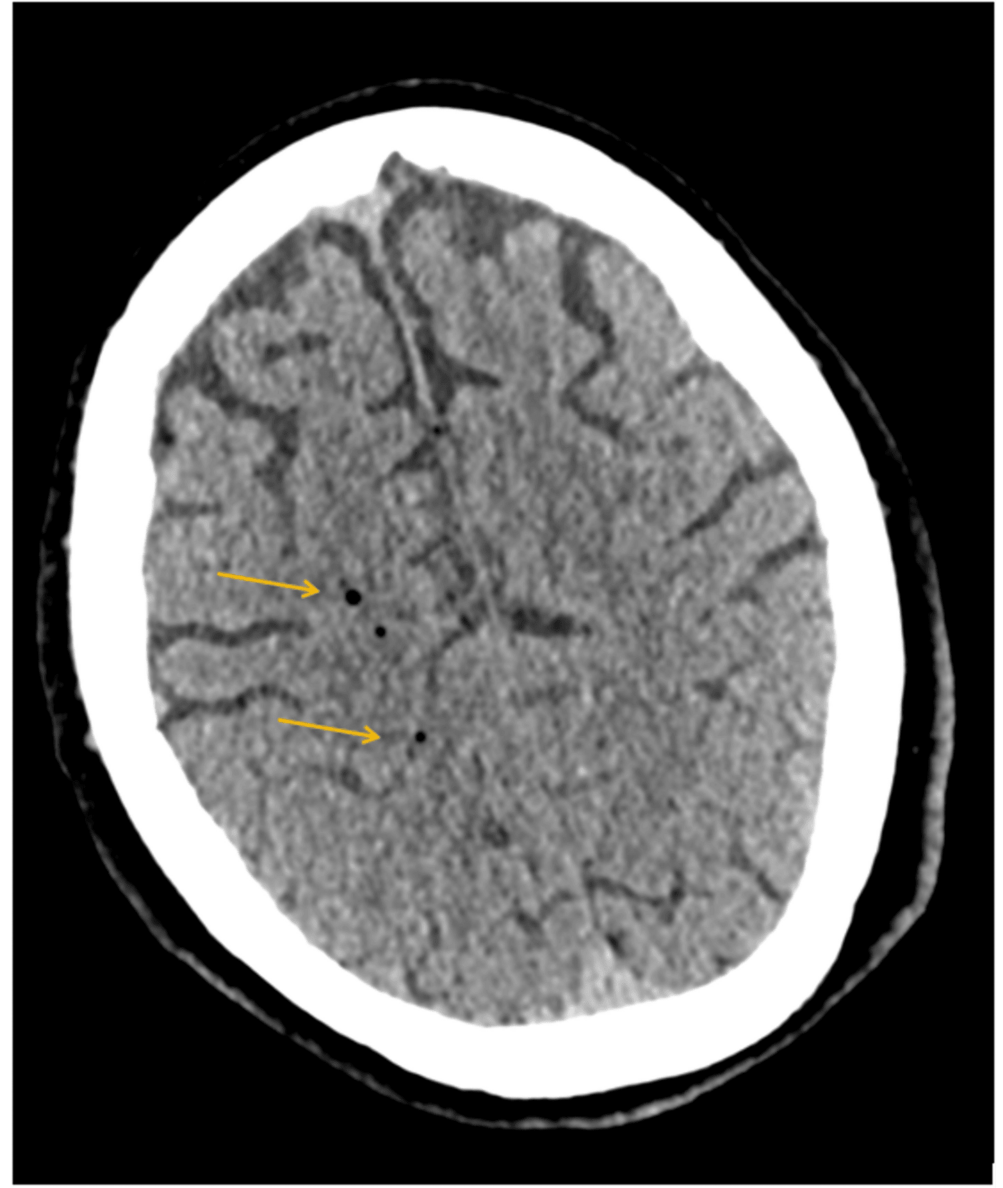
CT of the head axial non-contrast demonstrates a few small gas locules in the right centrum semi-ovale in keeping with air emboli. CT: computed tomography

**Figure 2:**
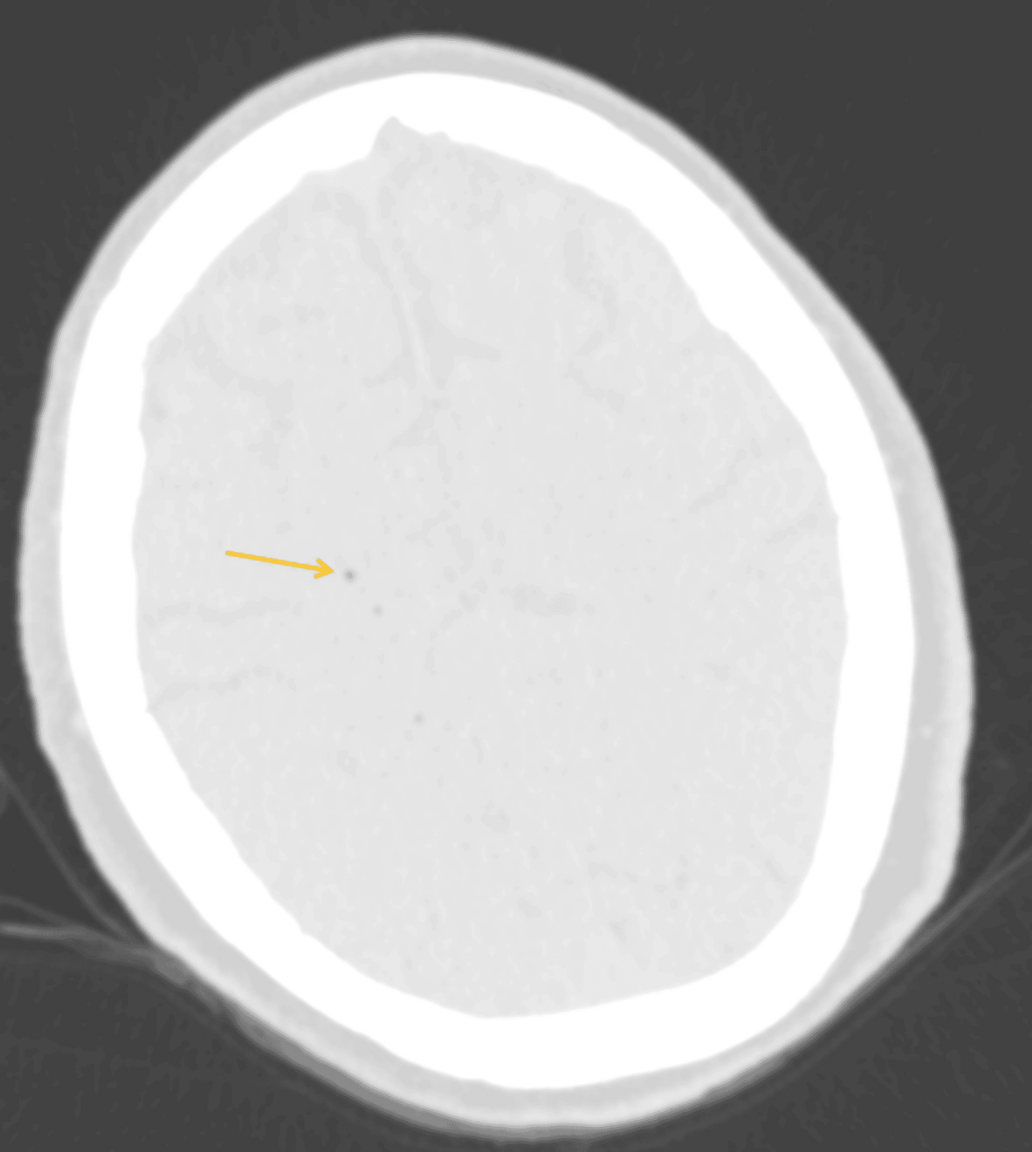
CT of the head axial non-contrast on lung windows allows for better visualization of the gas locules. CT: computed tomography

**Figure 3:**
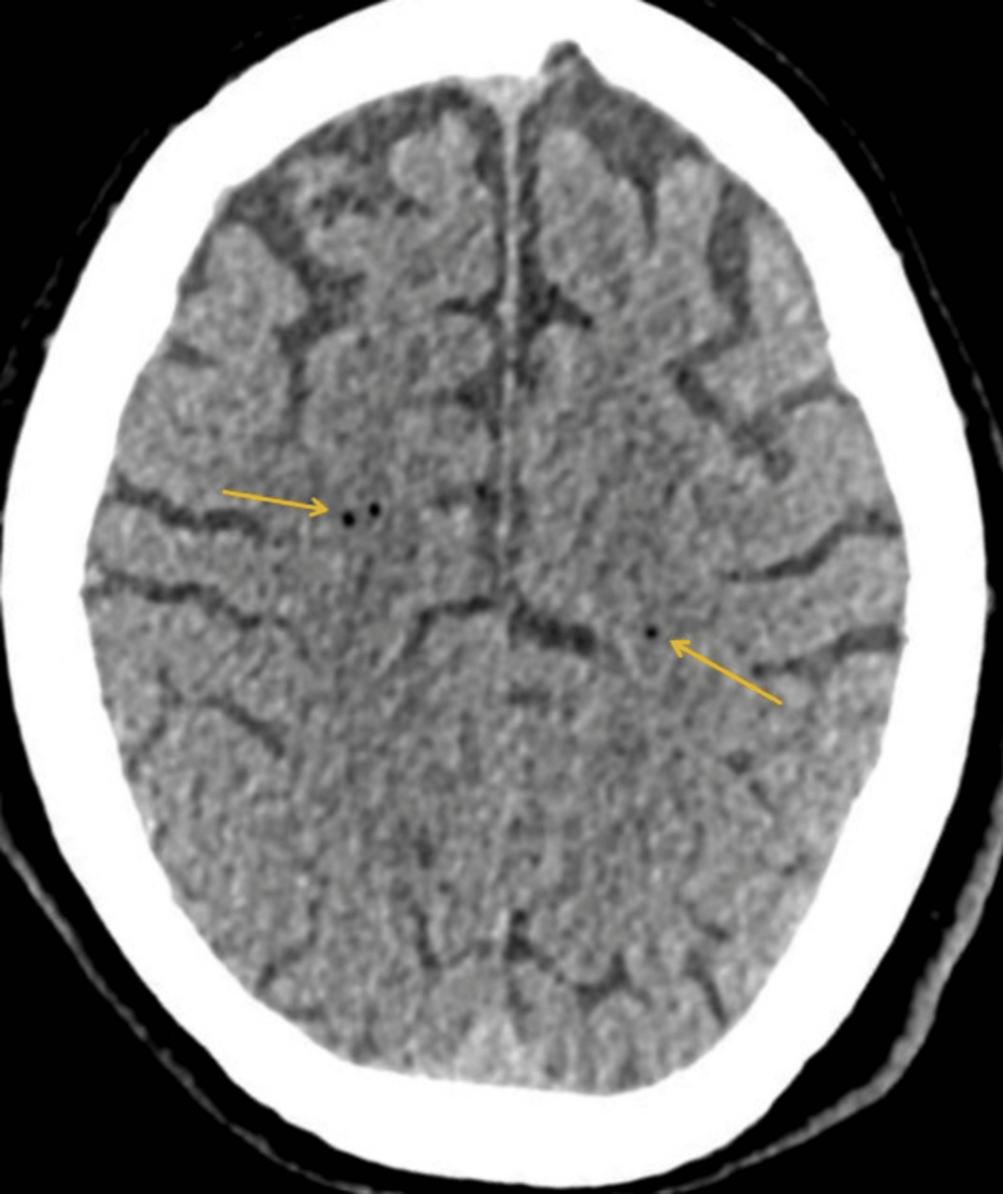
CT of the head axial non-contrast demonstrates a few small gas locules in the right centrum and the left semi-ovale in keeping with air emboli. CT: computed tomography

Around 12 days post-midline insertion, the patient developed sudden sensorimotor weakness on the left side of the body. A cerebral injury was suspected, and an initial computed tomography (CT) of the head showed an air embolus in the bilateral centrum semiovale (Figures 1-3). The centrum semiovale is a paired mass of white matter superior to the lateral ventricles. It plays a crucial role in communication within the brain as it acts as a bridge and can be responsible for functions related to language, memory, and sensory perception.

**Figure 4:**
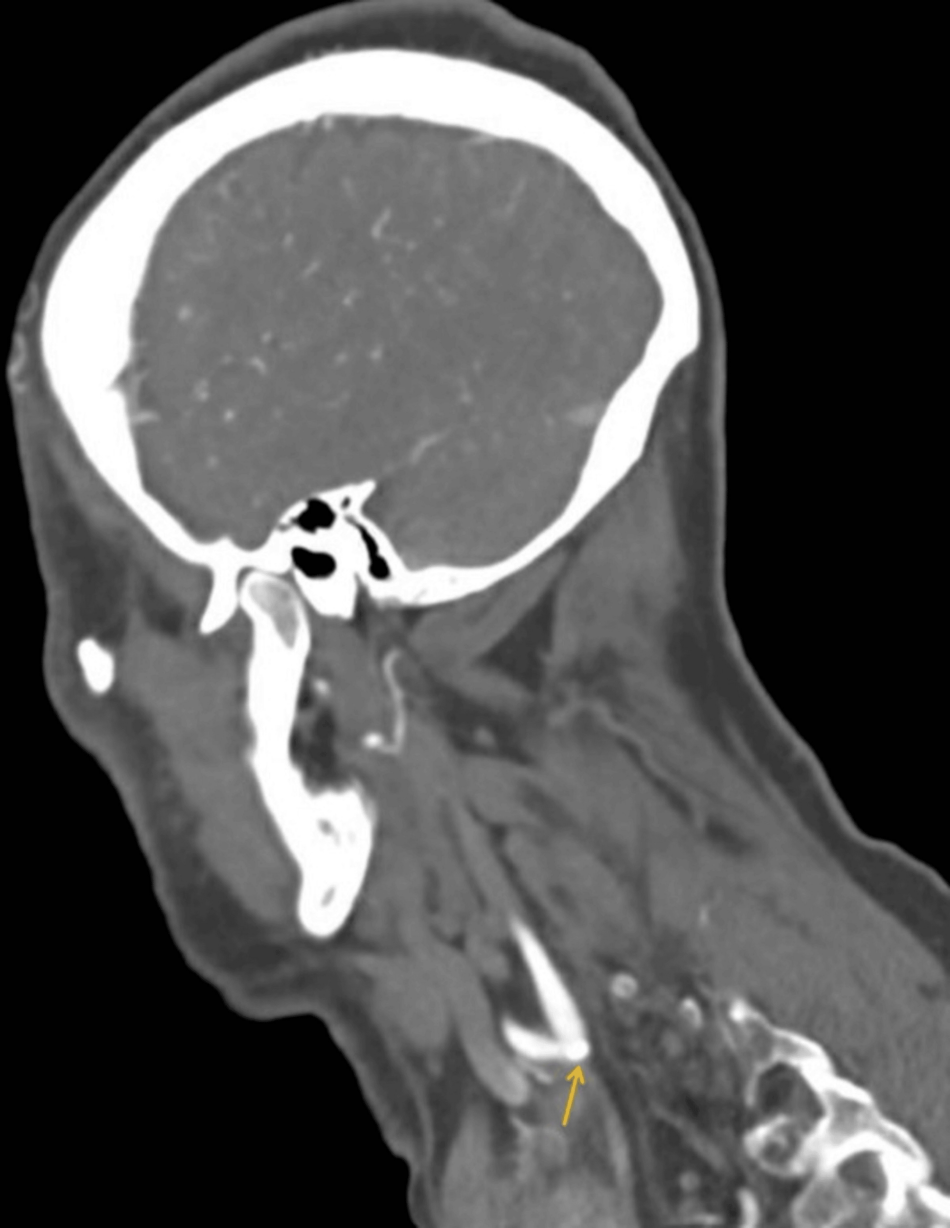
CT carotid angiogram sagittal section showing mild calcified atherosclerotic plaque just at the left carotid bifurcation. No significant intracranial stenosis or occlusion can be seen. CT: computed tomography

Further imaging with contrast-enhanced carotid angiogram revealed calcified atherosclerotic plaque without significant stenosis and no ischemic changes (Figure 4).

**Figure 5:**
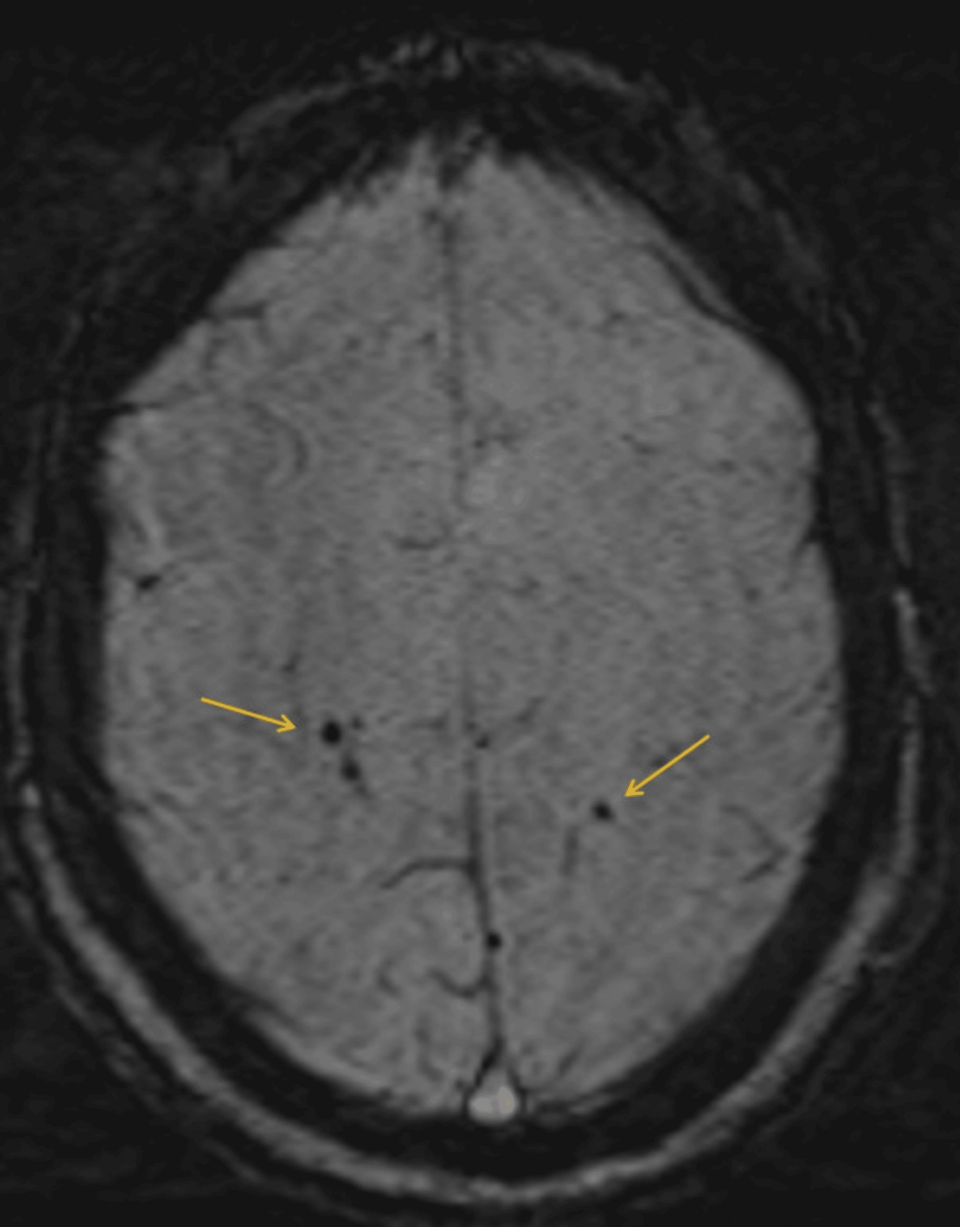
SWI on MRI of the head axial view demonstrates multiple corresponding focal areas of signal drop without a blooming artifact. No ischemic changes can be seen on the MRI. SWI: susceptibility-weighted imaging; MRI: magnetic resonance imaging

Further imaging with magnetic resonance imaging (MRI) of the head confirmed the presence of multiple small areas of air emboli without major ischemic changes (Figure 5).

After a discussion, the patient was under the care of the respiratory team at the time of the incident. The respiratory team raised suspicion that air may have entered the bloodstream when the patient received his dose of antibiotics through his midline catheter that day, as his symptoms began several minutes to hours after the antibiotic administration. Therefore, an incident report was filed.

Due to the comorbid COPD, our patient was not a suitable candidate for hyperbaric oxygen therapy as COPD patients are at an increased risk of developing type two respiratory failure when exposed to high oxygen levels that can lead to hypercapnia symptoms such as an increase in shortness of breath, disorientation, and possible confusion or altered mental state. Treatment was considered with 50% oxygen therapy to manage the air embolism. The midline catheter was suspected as a potential cause and subsequently removed to prevent further complications. No signs of damage were found during the inspection of the midline catheter itself. No local complications were noted such as subcutaneous emphysema, or even local hematoma.

The patient was transferred to the stroke rehabilitation ward. An initial assessment of day two of the stroke showed ongoing left upper and lower limb weakness, with improvement noted over time. By day three, he exhibited good recovery from stroke symptoms, although residual left-sided weakness persisted, particularly in the left upper limb. Follow-up assessments indicated mild proximal weakness in the left upper and lower limbs, with power rated at 4/5 on the Oxford muscle strength grading scale, and some sensory loss.

The patient was discharged on day 14 post-stroke and continued to receive support and physiotherapy in the community. Despite the challenges, the patient regained functionality and mobility, though assistance with daily activities such as washing and dressing was required.

An outpatient echocardiogram was performed a month after his discharge to rule out a patent foramen ovale (PFO). However, the operator was unable to definitively rule out PFO due to poor visualization caused by lung interference, as the extent of the patient’s lung disease made it difficult to assess the heart.

**Figure 6:**
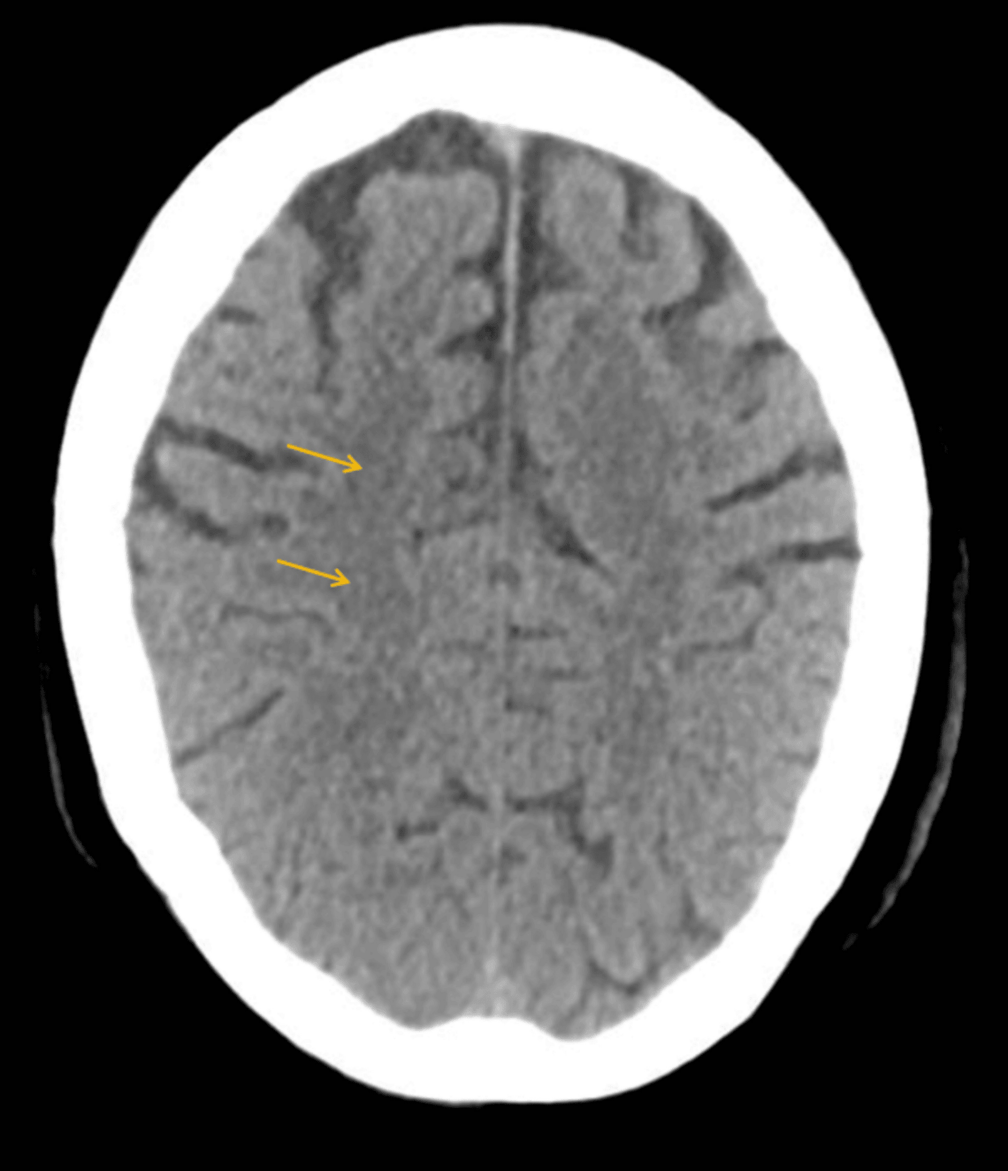
Complete resolution of the previously observed cerebral air emboli in Figure 1.

Over a month later, he was re-admitted with shortness of breath. A non-contrast CT of the thorax was requested due to multiple chest infections not responding to antibiotics courses. CT revealed a possible filling defect, raising suspicion of a pulmonary embolism. While awaiting a formal CT pulmonary angiogram scan (CTPA), the patient was started on treatment-dose tinzaparin. On the following day, he developed a new left-sided weakness. An urgent CT of the head was performed to exclude a cerebral bleed, which showed no hemorrhage and complete resolution of the previously observed cerebral air emboli (Figure 6). The patient also underwent a contrast-enhanced CTPA scan which showed no signs suggestive of pulmonary embolism.

Given his low Glasgow Coma Scale (GCS) score of 6, a decision was made to initiate palliative care. The patient passed away approximately one month later, suspected of having suffered a massive stroke that was not further investigated.

## Discussion

CAE can result from a variety of causes, such as trauma, surgical procedures, mechanical ventilation, barotrauma, and venous or arterial catheterization, including central and peripheral venous catheters such as the use of a midline catheter in this case [5]. The European Consensus Conference on Hyperbaric Medicine recommends using hyperbaric oxygen therapy for arterial and venous gas embolism presenting with neurological or cardiac symptoms. It is preferable that hyperbaric oxygen therapy be administered within the first four to six hours after symptom onset but continued benefit has been reported when hyperbaric oxygen therapy is delayed for up to 30 hours, as air entering the bloodstream due to compromised vessels or pressure gradient variations can block blood flow in the venous or arterial systems, leading to ischemic injury [6]. Hemodialysis has been linked to CAE, especially when air enters via central venous access during dialysis [7].

The neurological manifestations of CAE are remarkably comparable across cases as patients who underwent catheter implants or surgical procedures have reported similar abrupt-onset focal deficits, seizures, and altered consciousness. In many cases, such as this one where the diagnosis of CAE was made 12 days after the catheter was inserted, the diagnosis is still ambiguous. Although CAE has been linked to several different causes, it is still a rare disorder with a high mortality rate [8].

CAE symptoms usually manifest as an abrupt neurological impairment, convulsions, or loss of consciousness. This is exemplified in a case where a patient became unresponsive following a surgical procedure. Confusion and seizures are frequently observed, and a poorer prognosis is often associated with certain imaging findings. Symptoms may be delayed; in one case, a patient developed CAE while receiving therapy for congestive heart failure. Regular medical treatments may be a contributing factor to CAE. A case has been documented where several hemodialysis sessions following the removal of central venous catheters resulted in a deterioration of GCS and stroke-like symptoms [9].

Air trapped in a pyriform pattern in the context of CAE prognosis has been associated with poorer outcomes. Air emboli can manifest in multiple vascular regions, including the ophthalmic, carotid, and cortical vessels, adding to the complexity of diagnosis and treatment. Therefore, imaging techniques such as CT and MRI can promptly detect air in the brain parenchyma or vascular system making them crucial in the diagnosis of CAE [10].

One study highlighted the occurrence of CAE after laparoscopic surgery, where a CT scan of the brain revealed extensive air in the subarachnoid spaces and both cerebral hemispheres, consistent with a massive cerebral arterial air embolism. A follow-up CT demonstrated severe brain swelling, underscoring the challenges in managing severe cases of CAE [11].

A study demonstrated that CAE can also involve the cerebral venous sinuses, including the cavernous, superior sagittal, and straight sinuses, further illustrating the varied distribution of air emboli within the brain’s vascular system [1]. This distribution pattern adds another layer of complexity to CAE management and highlights the importance of comprehensive imaging in identifying all affected areas. Although CT scans are highly effective for immediate diagnosis, follow-up MRI scans provide a clearer picture of the ischemic changes caused by CAE. For example, in a recent case study, initial CT imaging showed air embolism, while an MRI performed later detailed the full extent of cerebral ischemia [12]. This approach highlights the importance of both early and continued imaging in managing and understanding the progression of CAE.

Management of CAE focuses on preventing further air emboli and minimizing brain damage. A systemic review and meta-analysis emphasized that the effectiveness of hyperbaric oxygen therapy decreases with delayed treatment, underscoring the importance of early therapy for optimal outcomes in CAE. There is a risk of using anticoagulant therapy such as aspirin and clopidogrel in CAE; however, there is not enough evidence to suggest an overall improvement. They might help prevent additional clotting around the air bubble, theoretically [8]. In a recent cohort study, death was noted as a significant outcome, with most patients experiencing minor to severe disability after CAE [11].

## Conclusions

CAE is a rare and severe condition that can arise from several causes, including medical procedures and complications related to surgical procedures, hemodialysis, and central venous access. CAE requires quick recognition and an immediate response for effective management. Sudden neurological symptoms or changes in consciousness levels after procedures should prompt urgent imaging, such as CT scans for initial detection and MRI scans for assessing further damage. Early rehabilitation is essential for functional recovery.

In cases where CAE occurs, early hyperbaric oxygen therapy often improves outcomes by reducing air bubble size and promoting absorption. Addressing the root cause of the air embolism is also essential to prevent further issues, especially as comorbidities may influence treatment options. The prognosis varies widely, as some patients recover fully while others may face long-term disabilities. Regularly reviewing case studies and recent research helps continually improve our understanding and treatment of CAE.
